# Memory Synapses Are Defined by Distinct Molecular Complexes: A Proposal

**DOI:** 10.3389/fnsyn.2018.00005

**Published:** 2018-04-11

**Authors:** Wayne S. Sossin

**Affiliations:** Department of Neurology and Neurosurgery, Montreal Neurological Institute, McGill University, Montreal, QC, Canada

**Keywords:** AMPA receptors, Aplysia, engram neuron, protein kinase M (PKM), synapse formation, synapse diversity, synaptic tagging and capture hypothesis, engram cells

## Abstract

Synapses are diverse in form and function. While there are strong evidential and theoretical reasons for believing that memories are stored at synapses, the concept of a specialized “memory synapse” is rarely discussed. Here, we review the evidence that memories are stored at the synapse and consider the opposing possibilities. We argue that if memories are stored in an active fashion at synapses, then these memory synapses must have distinct molecular complexes that distinguish them from other synapses. In particular, examples from *Aplysia* sensory-motor neuron synapses and synapses on defined engram neurons in rodent models are discussed. Specific hypotheses for molecular complexes that define memory synapses are presented, including persistently active kinases, transmitter receptor complexes and trans-synaptic adhesion proteins.

## Memories are stored at synapses

Most neuroscientists believe that memories are encoded by changing the strength of synaptic connections between neurons (Mayford et al., 2012; Poo et al., 2016). The great success of deep learning systems based on units connected by modifiable synaptic weights has greatly increased the confidence that this type of computational structure is a powerful paradigm for learning. Nevertheless, the question of whether memories are stored locally at synapses remains a point of contention. Some cognitive neuroscientists have argued that for the brain to work as a computational device, it must have the equivalent of a read/write memory and the synapse is far too complex to serve this purpose (Gaallistel and King, 2009; Trettenbrein, 2016). While it is conceptually simple for computers to store synaptic weights digitally using their read/write capabilities during deep learning, for biological systems no realistic biological mechanism has yet been proposed, or in my opinion could be envisioned, that would decode symbolic information in a series of molecular switches (Gaallistel and King, 2009) and then transform this information into specific synaptic weights. Until such a mechanism is proposed and tested, it is reasonable to assume that there are specific changes at the synapse that serve to store the changes in synaptic weights that underlie memory.

The above is not an argument that neuron-wide changes do not play an important role in memory formation and maintenance. Indeed, it is clear that when “engram” neurons—so-called because they embody the trace that experience has left on the brain—are allocated to the memory trace, they activate a program of gene expression in the nucleus (Kandel et al., 2014; Liu et al., 2014). The change in gene expression in engram neurons leads to cell-wide changes in the neuron independently of synaptic changes. One clear example of these changes is transient increase in the excitability of the neuron (Zhou et al., 2009; Yiu et al., 2014). The change in excitability has the consequence of linking memories of events occurring close together in time (Cai et al., 2016; Kastellakis et al., 2016). In some systems, an increase in excitability plays an important role in encoding memory (Mozzachiodi and Byrne, 2010; Titley et al., 2017). However, the computational power of cell-wide changes is quite limited and in my judgment, it seems unlikely that complicated learning algorithms could be implemented using only cell-wide changes.

The selection of a neuron to participate in a memory may also leave long-lasting transcriptional marks such as changes in histone and DNA methylation, and long-term changes in the organization of the nucleus (Heyward and Sweatt, 2015; Cholewa-Waclaw et al., 2016; Sweatt, 2016; Watson and Tsai, 2017). Indeed, there is evidence that changes in transcription are not only important for learning, but continued transcriptional changes are important in the maintenance of memory and thus, removal of transcriptional marks can erase memory (Miller et al., 2010; Pearce et al., 2017). Further, even after synaptic expression of memory has been erased, transcriptional marks can lead to changes in the neuron's ability to induce later memory (Chen et al., 2014; Pearce et al., 2017). Thus, transcriptional marks lead to a form of “savings” that is truly synapse-independent. While the above demonstrations from the Glanzman lab have been used to argue that memories are, indeed, not stored at synapses (Poo et al., 2016; Trettenbrein, 2016), they are also consistent with a model whereby long-term transcriptional marks encode changes in engram neurons that work together with synaptic mechanisms for storage of specific weights locally at synapses to encode memory. As mentioned above, for transcriptional marks to be independent of synaptic changes, such marks would be required to “know” which specific connections will require strengthening, and in my opinion there is no plausible model by which this can occur in the absence of some synapse-specific memory mechanism.

The concept that synapse-specific changes need to interact with products of gene expression to express memory at specific synapses is similar to the model of synaptic tagging. In models of synaptic tagging, the learning stimuli result in the formation of tags at activated synapses that then capture the products of gene expression in order to change the strength of the synapse (Frey and Morris, 1997, 1998a; Martin et al., 1997). A number of molecular models for the synaptic tag have been proposed including changes in actin polymerization, AMPA receptor insertion, protein kinases and specific adaptor proteins (Martin and Kosik, 2002; Sajikumar et al., 2007; Redondo et al., 2010; Moncada et al., 2011).

In synaptic tagging models, the products of gene expression have effects only on the activated synapses with tags. The ability to dissociate the setting of the tag from the activation of gene expression has allowed powerful tests of these models (Frey and Morris, 1998b; Casadio et al., 1999; Frey and Frey, 2008). This model has also been supported in behavioral experiments where weak learning can be transformed into stronger learning by a separate experience that activates gene expression (Moncada and Viola, 2007). This phenomenon has been termed behavioral tagging (Viola et al., 2014; Moncada et al., 2015).

If transcription were also required for the maintenance of memory, it would suggest that a form of synaptic tagging may also be important for the maintenance of memory. However, in synaptic tagging the half-life of the tag has been measured to be at most a few hours (Martin et al., 1997; Frey and Morris, 1998b), whereas the half-life of the tag would need to be much longer for it to play a role in the maintenance of memory. While in the model of synaptic tagging, the tag is made independently of gene expression, a similar model for the maintenance of memory would probably involve a role for transcription in maintaining the tag. One way of thinking about distinct molecular complexes present at memory synapses is that these complexes act as long-lasting synaptic tags enabling long-lasting maintenance of synaptic modifications. In the absence of the transcriptional marks and the ongoing production of proteins important for the maintenance of these complexes, the molecular complexes underlying the maintenance of memory-specific synaptic changes may fall apart explaining the requirement for ongoing transcription to maintain long-term synapse-specific changes.

The other major recent finding that has been used as an argument against memories being stored at synapses comes from the Tonegawa lab (Ryan et al., 2015; Roy et al., 2017). Tonegawa and his colleagues found that when retrograde amnesia was induced by adding protein synthesis inhibitors shortly after learning, synaptic changes onto engram cells were blocked and re-exposure to the learned stimulus did not lead to recall. However, the memory could still be recalled by optogenetic activation of the “silent engram” cells. While this appears to show that memories are stored independently of synaptic changes, the alternative storage mechanism proposed by the authors depends on the increased connections made between the engram cells even in the presence of the protein synthesis inhibitors (Poo et al., 2016). However, these increased connections are likely still due to synaptic changes. Thus, in my view, the findings do not suggest that synaptic changes do not encode memory, but rather that distinct synaptic changes encoding memory show differential sensitivity to protein synthesis inhibitors. Moreover, these studies do not show that memory formation does not require protein synthesis, since the experiments require optogenetic and fluorescent markers to be produced in the engram cells as a consequence of experience-driven protein synthesis (Ryan et al., 2015). Thus, enough protein synthesis was induced to make the marker proteins and some synaptic changes, but not enough for the synaptic changes that allow the sensory stimulus to activate the engram cells. In my opinion, this finding is evidence for diversity in encoding of memory synapses and will be discussed further in the last section of the review.

## Defining a memory synapse

### Distinct synapses have different functions

Due to differential gene expression, the synapses of distinct neurons have distinct properties (Grant, 2007; O'Rourke et al., 2012). There are many examples where neuronal synapses are optimized for a certain function. For example in the auditory system, where following accurately at high frequency is particularly important, neurons have specialized synapses with unique properties that allow for this function (Wichmann, 2015). Neurons can express different receptor subunits (Cull-Candy and Leszkiewicz, 2004; Greger et al., 2017), receptor accessory proteins (Kato et al., 2010; Greger et al., 2017), synaptic adhesion proteins (de Wit and Ghosh, 2016), calcium channels (Kamp et al., 2012), active zone proteins (Mittelstaedt et al., 2010; Südhof, 2012; Crawford and Kavalali, 2015; Torres and Inestrosa, 2017) and scaffold proteins (Emes and Grant, 2012). This diversity is not only important for differentiating properties of different neurons, but also allows individual neurons to have diversity between their synapses (de Wit and Ghosh, 2016; Yamasaki, 2016; Zampini et al., 2016). In my view, the memory synapse would be just another example of a specialized synapse, with a specific complement of receptor complexes, synaptic adhesion proteins, active zone proteins and scaffold proteins defining the specific properties of a synapse that supports memories. In particular, a memory synapse would have specializations that allow for sensitivity to memory-altering stimulation but stability to other perturbations.

### Memory erasure provide the evidence for specialized memory synapses

The idea that specific memory synapses exist stems from evidence that memories can be specifically erased without affecting general synaptic connectivity. If the synaptic connections underlying memory can be specifically erased without disturbing other synaptic connections present in the same circuit or brain region, logically, there must be distinct molecular complexes at these synapses that explain the sensitivity to memory erasers. Other synapses that are not affected under these conditions would therefore lack these distinct molecular complexes. This idea is distinct from deep learning, where there are no privileged synaptic connections that underlie memory; all connections are equally modifiable. It is possible that in the brain, while memories are stored at synapses, encoding is due to a reweighing of all synaptic weights over a local circuit, similar to deep learning paradigms. In this case, there would be no specific “memory” synapses. However, such models are contradicted by the evidence, summarized in the next few sections, that erasure of memory only affects synapses modified by learning. If this evidence is to be believed, then logically, there must be molecular differences at memory synapses that allow them to be differentially affected when memories are specifically erased.

#### Memory erasure by reconsolidation

The first indication that memory could be specifically erased comes from the effects of blocking reconsolidation. Recalling a memory makes it labile and when inhibitors of gene expression or protein synthesis are used after recall, the memory is erased (Nader et al., 2000). This property is thought to be important for the ability to adapt to changing circumstances, i.e., to update memory based on new information (Lee et al., 2017). In contrast, simple activation of a circuit is not sufficient to induce lability of the circuit. Perception of sensory stimuli, such as light and sound, involves repetitive activation of synapses that do not become labile to reconsolidation every time a sensory stimulus occurs. The effects of blocking reconsolidation suggest that the synapses underlying memory are fundamentally different from the synapses underlying perception. It could be argued that reconsolidation represents a cell-wide property, not a synaptic one, similar to ideas expressed above regarding transcriptional marks and changes in excitability. However, even if reconsolidation only occurs in “memory neurons,” not all synapses in that neuron are removed. Another possibility is that the loss of memory after a blockade of reconsolidation represents a circuit property and does not represent a reversal of synaptic changes underlying memory. For example, behavioral extinction can also erase the expression of a memory and there is a general consensus that this erasure is due to additional circuit changes (new learning) and not the reversal of the original memory (Tovote et al., 2015). Answering the question of whether the blockade of reconsolidation removes synaptic changes underling memory will require specific examination of the synapses that changed in strength after learning and comparison of these synapses to other synapses present in the neuron after the blockade of reconsolidation. This has not yet been done in vertebrate neurons, although recent advances in the identification of engram cells and the ability to specifically monitor changes in synaptic strength associated with memory formation suggests that it could be done in the near future. Indeed, a recent study showed that synaptic changes in engram neurons were not reduced by extinction (Kim and Cho, 2017), consistent with extinction being distinct from erasure. There is also some evidence for decreased synaptic input after reconsolidation (Doyere et al., 2007; Diaz-Mataix et al., 2011), consistent with the erasure of memory synapses by reconsolidation, but this result is mainly correlative. Later, we will discuss the evidence from a reductionist model in *Aplysia* that reconsolidation specifically erases the changes induced by synaptic plasticity.

#### Memory erasure by pharmacology and dominant negative constructs

Erasure of memory has also been observed, even in the absence of recall, through interference with persistent protein kinases (using either pharmacological agents or dominant negative constructs of persistently active kinases; Rossetti et al., 2017; Sacktor and Hell, 2017). In vertebrates such experiments do not specifically measure changes in synapses that are increased by learning, similar to the reconsolidation studies discussed above. However, in the reductionist *Aplysia* system, there is strong evidence that inhibitors of persistent protein kinases specifically remove memory synapses without affecting basal synaptic strength (Cai et al., 2011; Hu et al., 2017a); these experiments are described in detail below. Although there is considerable controversy over the specificity of such inhibitors and dominant negatives (Lisman, 2012; Wu-Zhang et al., 2012; Yao et al., 2013; Tsai et al., 2015; Farah et al., 2017), the evidence that these reagents specifically erase memories without altering the overall circuit or affecting relearning can be most easily explained, in my judgment, by the presence of distinct molecular complexes at memory synapses that are targeted by these memory erasers. Notably this conclusion is independent of the actual identity of the targets of these reagents.

#### Memory erasure with optogenetics

Memory erasure has also been accomplished in vertebrate systems by optogenetically inducing long-term depression (LTD) in the inputs to engram neurons (Nabavi et al., 2014; Kim and Cho, 2017; Klavir et al., 2017). One interpretation is that LTD can also specifically erase the memory synapse, but these experiments lack evidence that LTD only decreases synaptic strength at memory synapses, as opposed to decreasing synaptic strength at any or all connections. There is some evidence that certain LTD protocols, particularly mGLUR-LTD, are limited to decreasing synaptic strength at those synapses that have previously undergone LTP and may thus represent memory synapses (Jones, 2017). However, the ability to induce forms of LTD at many synapses suggests that LTD is not strictly limited to memory synapses. The important question becomes whether the molecular mechanisms underlying a particular type of LTD can remove a particular type of molecular memory complex; this is discussed further below in the context of AMPA receptor complexes.

## Insights about memory synapses from the reductionist *Aplysia* sensory-motor neuron system

### The sensory-motor neuron system

Sensitization in *Aplysia* is defined as an increased defensive reflex in response to an innocuous stimulus after a noxious stimulus. Sensitization is induced by the release of serotonin in response to a noxious stimulus (Glanzman et al., 1989; Marinesco et al., 2004). Repeated noxious stimulation leads to long-term increases in the defensive reflex (Bailey and Chen, 1989). Long-term sensitization can also be associative when touch to the animal is paired with the noxious stimulus, and in this case there is an increase in the response to the touched area (Hawkins, 1984). The behavioral memory of long-term sensitization is stored in part by increases in the strength of the connection between touch-sensitive sensory neurons and motor neurons important for defensive withdrawal (Kandel, 2001). A major advantage of this system is that the synaptic changes induced during memory formation can be recapitulated after sensory and motor neurons are removed from the animal and cultured (Montarolo et al., 1986). The ability to access long-term synaptic changes induced with stimulation in culture very similar to the induction process in the animal has allowed detailed investigation of the molecular steps involved in generating a long-term memory trace. Several of the original findings in this system, such as a requirement for cAMP signaling and CREB activation in the initial induction of long-term synaptic changes, have been shown to be universal, further enhancing the utility of this system (Bailey et al., 1996).

The long-term increase in synaptic strength that is linked to long-term memory in *Aplysia* is called long-term facilitation (LTF). A number of additional characteristics of LTF in *Aplysia* are relevant to the discussion of a memory synapse: (1) the distinct stages in long-lasting LTF that are dependent on time and the number of training applications; (2) the role of new synapse formation in LTF; and (3) the differences between associative and non-associative LTF. These characteristics are summarized briefly here and will be explored in more detail below with the description of erasing memory complexes in this model.

#### Distinct stages in long-lasting LTF

Non-associative LTF induced by five spaced applications of serotonin (5HT) in sensory motor neuron cultures lasts approximately 3 days and is maintained by distinct phases. For the first 12 h (h), maintenance depends on persistent activation of protein kinase A (PKA), but this dependence disappears by 24 h (Hegde et al., 1997; Chain et al., 1999). Many transcriptional changes are observed at 24 h that are not observed at 1 h (Conte et al., 2017), suggesting that there are waves of transcriptional and translational products made after learning and consistent with the idea that distinct molecular complexes underlie memory at different times after training (Sossin, 2008). Multiple training sessions can produce longer lasting sensitization (Bailey and Chen, 1989) and multiple days of 5HT application lead to longer-lasting LTF of at least 7 days (Hu et al., 2011). The observation that there are distinct synaptic properties present after two training sessions that are not present after single sessions, such as changes in presynaptic depression rate in associative LTF (Hu and Schacher, 2015; Hu et al., 2017b), suggests that multiple training sessions may also affect the molecular complexes that retain memory. Indeed, the concept that the molecular complex underlying memory is changed over a time period of days is an important one that has not received much attention. For example, is the molecular complex that retains memory after reconsolidation (activation followed by a second protein synthesis-dependent consolidation) the same as the molecular complex formed after a single consolidation event?

#### The role of new synapses in long-lasting LTF

While new synapses are observed in cultures of sensory-motor neurons after induction of LTF, they are not observed in the animal in the absence of multiple training sessions over multiple days (Wainwright et al., 2002). Moreover, the amount of LTF seen at 24 h is not significantly different when new synapses are not generated, for example when serotonin application is restricted to the cell body (Sun and Schacher, 1998; Casadio et al., 1999). Although the new synapses correlate well with long-lasting LTF, there is no conclusive evidence that LTF occurs only at the new synapses, as opposed to being stored mainly in old synapses or in a combination of new and old synapses. Whether memory synapses are generated from new synapses is a major unanswered question in the field.

#### Non-associative vs. associative LTF

Similar to LTP in vertebrates, there are multiple distinct stimuli that can generate LTF. First, for non-associative LTF (LTF generated in the absence of action potentials in the sensory or motor neuron), differences in timing of 5HT application lead to LTF that lasts for distinct periods of time (Smolen et al., 2016; Kukushkin and Carew, 2017). Associative LTF can be generated by pairing action potentials in the sensory neuron with one application of 5HT (Hu et al., 2017a). As described below, the molecular complex that maintains increases in synaptic strength is different after associative and non-associative LTF.

### Erasing memory synapses by blocking reconsolidation in *Aplysia*

Behavioral sensitization in *Aplysia* is lost when reconsolidation is blocked using protein synthesis inhibitors after a reminder (Cai et al., 2012; Lee et al., 2012). In this system the question of whether the synaptic changes that occurred during learning are reversed when reconsolidation is blocked can be addressed directly, since the molecular changes that occur during behavior can be recapitulated in culture. Indeed, LTF of sensory-motor synapses is reversed in sensory-motor neuronal cultures when protein synthesis inhibitors are added to the cultures after a reminder (either firing the sensory neuron, or adding one pulse of serotonin in cultures; Cai et al., 2012; Lee et al., 2012; Hu and Schacher, 2014). In three independent experiments from different labs, blockade of reconsolidation did not remove all synapses between the cultured sensory neuron and motor neuron, but instead reduced synaptic strength back to the basal level seen before inducing LTF (Cai et al., 2012; Lee et al., 2012; Hu and Schacher, 2014). This finding is consistent with the erasure of a subset of synapses that could be defined as memory synapses. Follow-up experiments from the Glanzman lab have suggested that the loss of synaptic strength is correlated with a loss of physical synapses, but that the loss is not specific to the new synapses generated by facilitation (Chen et al., 2014). While this result can be interpreted as evidence that changes in synaptic strength are not stored at synapses, an alternative explanation is that under these circumstances, memory synapses are not derived mainly from new synapses.

### Erasing memory synapses with pharmacological and dominant negative constructs in *Aplysia*

The zeta inhibitory peptide (ZIP) and chelerythrine are pharmacological agents that erase memories in vertebrates (Sacktor, 2011) and erase behavioral sensitization in *Aplysia* (Cai et al., 2011). These inhibitors also erase LTF in sensory-motor neuron cultures (Cai et al., 2011). The intended target of the inhibitors are truncated, persistently active protein kinase Cs (PKCs), known as protein kinase Ms (PKMs). In *Aplysia*, there are three PKCs present in the nervous system: the classical PKC Apl I (homologous to PKCα, β, and γ in vertebrates); the novel PKC Apl II (homologous to PKCε and η in vertebrates); and the atypical PKC Apl III (homologous to PKCι and ζ in vertebrates). PKMs made from any of the three PKCs are inhibited to almost equal extents by ZIP peptide and chelerythrine (Villareal et al., 2009; Farah et al., 2017).

To gain independent evidence that PKMs sustain long-lasting increases in synaptic strength and to determine if specific isoforms of PKM were involved in maintaining LTF dominant negative PKMs were expressed in sensory-motor neuron cultures. The dominant negative PKMs were generated with an aspartate to alanine mutation that has been shown to greatly reduce kinase activity, but to retain priming phosphorylation of the kinases, and thus to minimize non-isoform specific effects mediated by sequestering upstream kinases (Cameron et al., 2009; Bougie et al., 2012). Conformational changes induced by the priming phosphorylations may also be important in maintaining binding interactions important for localization of the PKMs and may be important for the ability of these reagents to work as isoform-specific dominant negatives.

In these experiments, erasure of memory was initiated after two stimuli separated by a day, and the dominant negatives were then expressed in the pre- or postsynaptic cell 2 days after the last stimulus. These dominant negatives reversed the increase in synaptic strength seen after LTF (Hu et al., 2017a,b). Strikingly, associative and non-associative LTF were decreased back to the level of synaptic strength seen before induction by distinct dominant negative PKMs (Hu et al., 2017a). Interestingly, LTF was reversed after injecting dominant negative PKMs into either the presynaptic or the postsynaptic cell (Hu et al., 2017a), suggesting that persistent kinases in both compartments are required for maintenance of the memory synapse. Importantly, these dominant negatives had no effect on the synaptic strength between sensory and motor neurons if LTF was not induced (Hu et al., 2017a). The specific decrease of facilitated increases in synaptic strength strongly supports a model where the molecular complexes at facilitated synapses are distinct from those supporting basal synaptic strength. The fact that distinct dominant negative PKMs erased LTF after either non-associative or associative LTF is most easily explained by postulating distinct molecular complexes requiring different PKMs to retain memory after distinct stimuli. Indeed, when non-associative and associative LTF were induced in two distinct inputs onto the same postsynaptic motor neuron, specific expression of dominant negatives in the postsynaptic neuron could erase one form of LTF, leaving the other intact (dominant negative atypical PKM Apl III blocked associative LTF, while dominant negative classical PKM Apl I blocked non-associative LTF, Hu et al., 2017b). Thus, the dominant negative PKMs could distinguish molecular complexes involved in associative and non-associative LTF, interfering specifically at synapses with one type of complex but not the other, and not interfering with other synapses between the presynaptic and the postsynaptic cell representing basal synaptic strength (non-memory synapses).

### Comparisons between *Aplysia* and vertebrate memory synapses

Associative LTF has similarities with late-LTP. They both require firing of the presynaptic neuron, NMDA receptors (Murphy and Glanzman, 1997), and activation of a biogenic amine pathway, which is dopamine for late-LTP (Frey et al., 1990). In *Aplysia*, the atypical PKM is required for the maintenance of associative LTF in the postsynaptic cell, similar to the requirement for its orthologue PKMζ for late-LTP (Ling et al., 2002; Serrano et al., 2005). Two findings from *Aplysia* have not yet been shown to apply to vertebrate neurons: (1) The requirement for presynaptic as well as postsynaptic PKMs; and (2) The requirement for PKMs other than the atypical PKMζ to maintain distinct memory complexes. These findings have not been examined in vertebrates because pharmacological agents do not distinguish between presynaptic and postsynaptic neurons, and experiments with dominant negatives in vertebrate cells have used viral transductions that affect both presynaptic and postsynaptic cells during memory maintenance. There is also no evidence for isoform-specificity of the dominant negatives used in vertebrate experiments; indeed, the mutation used leads to a loss of priming phosphorylation (Cameron et al., 2009) and may inhibit upstream activators common to all isoforms of PKM (Garcia-Paramio et al., 1998). Furthermore, a cellular analog for non-associative LTF has not been established in vertebrate neurons, and it is not clear what role this form of plasticity plays in vertebrate learning. These will be interesting questions to ask in the future.

Experiments in vertebrates have however provided evidence in support of different types of memory synapses. For example, distinctions between different types of memory synapses can be inferred in experiments from the Tonegawa lab looking at the addition of protein synthesis inhibitors after learning. In mice, adding protein synthesis inhibitors after learning blocked the formation of memory synapses between entorhinal cortex neurons and dentate gyrus neurons, while leaving increased connections between engram neurons in dentate gyrus and CA3 intact. Indeed, the initial forms of LTP in these two pathways are distinct, with synapses between dendate gyrus and CA3 (mossy fiber synapses) exhibiting mainly changes in presynaptic properties (Zalutsky and Nicoll, 1990). Thus, it is not surprising that the eventual memory synapses formed in these two distinct neuronal connections are different.

A major distinction between PKM formation in vertebrates and *Aplysia* is the ability to form a PKM by translation of an alternative transcript in vertebrates (Hernandez et al., 2003). This transcript is only found in vertebrates (Bougie et al., 2009). In *Aplysia*, calpain activation has been linked to the formation of PKMs (Farah et al., 2017; Hu et al., 2017a). In contrast, while calpain activity is required for the induction of LTP and memory in vertebrates (Amini et al., 2013; Baudry and Bi, 2016), the requirement for calpains has not been linked to cleavage of PKC. Moreover, the orthology between the classical calpain required for induction of associative LTF in *Aplysia* (Hu et al., 2017a) and vertebrate classical calpains is not obvious (Hastings et al., 2017). A complete knockout of PKC/PKM ζ in vertebrates has mild (Tsokas et al., 2016) or no (Lee et al., 2013; Volk et al., 2013) effects on memory, but vertebrates have two atypical PKCs, and PKCι can partially compensate for PKMζ in maintaining late-LTP and memory (Tsokas et al., 2016). How PKCι is persistently activated is not clear, but there are increased levels of phosphorylated PKCι after learning (Tsokas et al., 2016), and similar to CAMKII (Lisman, 2017a), a model for a positive feedback loop of phosphorylation has been proposed (Jalil et al., 2015). It may be that after evolving a mechanism for generation of PKM in the absence of calpain, the role of calpain in generating PKMs for memory has been lost in vertebrates. Alternatively, compensation for the loss of PKMζ may be due to cleavage of PKCι to PKMι by calpains, although this is purely speculative at present. Similarly, whether calpains cleave other isoforms of PKC in vertebrates to form PKMs important for memory maintenance, either in the presynaptic cell, or for other forms of plasticity, remains an open question.

## Are memory synapses new synapses?

**Figure 1:**
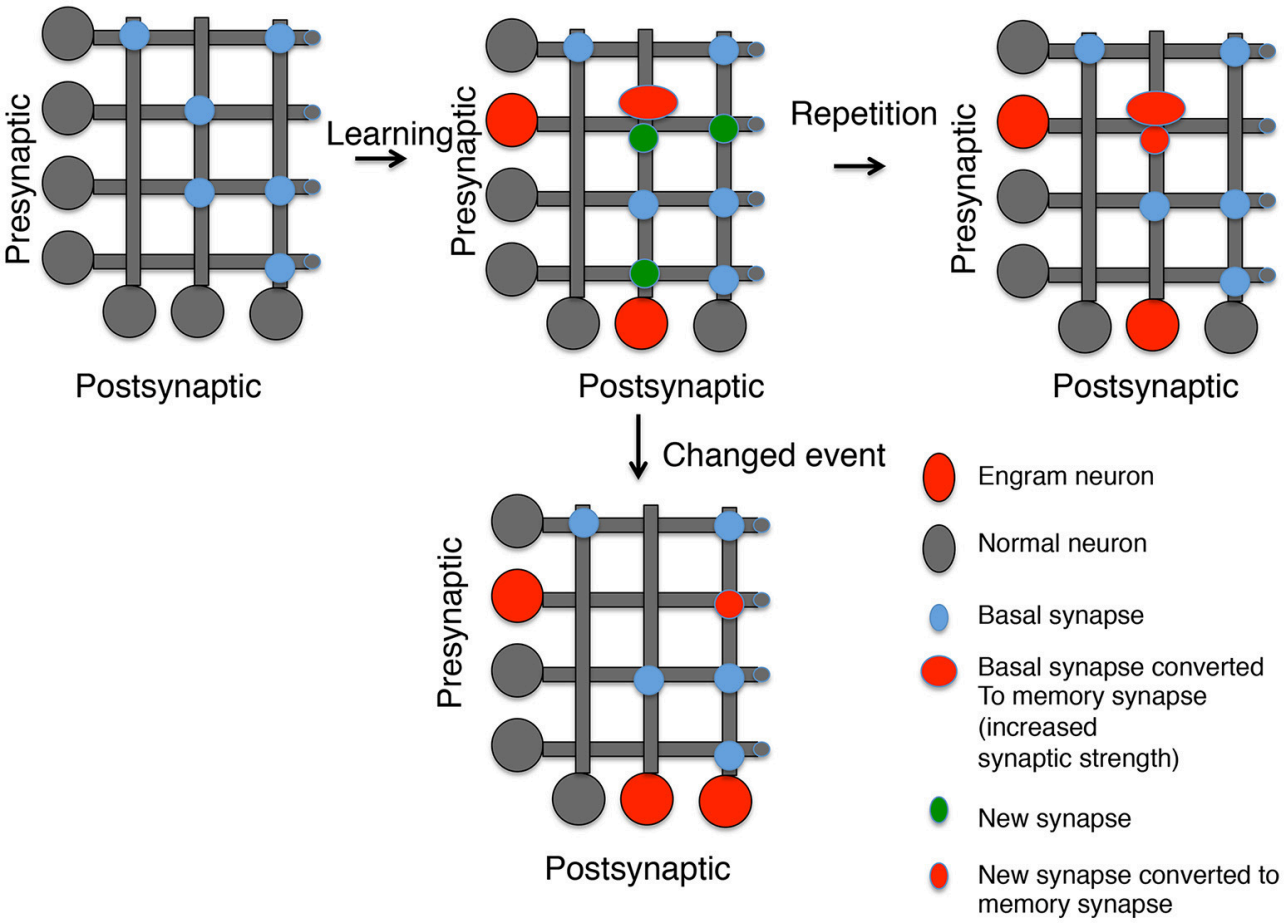
Generating memory synapses. A set of neurons is presented with distributed basal synapses between presynaptic and postsynaptic pairs. An initial learning stimulus will (1) Allocate the memory to engram neurons (red somas); (2) Convert basal synapses between engram neurons to memory synapses with increased synaptic strength; and (3) Generate new synapses, both to neurons previously connected by synapses and neurons not connected. A repetition of the same learning stimulus can convert the new synapses into memory synapses between engram neurons, but new synapses between other neurons decay. However, a similar stimulus that is now paired with a different experience can lead to (1) Generation of new engram neurons; (2) the erasure of previous memory synapses, and (3) the conversion of new synapses between the old engram and new engram neuron into memory synapses.

There is evidence from many systems that new synapses are formed after a memory stimulus. This includes the evidence discussed above from *Aplysia*, live imaging of spines after motor learning in rodents (Xu et al., 2009; Fu et al., 2012; Yang et al., 2014) and counting the number of spines in Engram neurons (Ryan et al., 2015). It is difficult, however, to know whether these new synapses are where memories are stored or whether they are a form of meta-plasticity providing a source of synapses that could become memory synapses in the future. We have argued that when a neuron is chosen as an Engram neuron it both strengthens inputs associated with the learning stimulus (tagged synapses) and generates new outputs to communicate the new information gained to new sources (Sossin, 1996). These new outputs would then be the source of new potential memory synapses when the memory is reactivated or associated with a new stimulus (Figure 1). This model is supported by studies in *Aplysia* where it has been shown, using a single day of stimulation, that new synapses are not specifically lost when a memory is erased (Chen et al., 2014). Studies examining spines also showed that the half-life of new synapses is in general less than a day (Trachtenberg et al., 2002; Holtmaat et al., 2005, 2006; Yang et al., 2014), but learning leads to increased stabilization of new synapses (Holtmaat et al., 2006; Yang et al., 2014; Li et al., 2017). Thus, an attractive model is that spaced learning events separated by days lead to memories by recurrent formation of new synapses, followed by conversion of the new synapses into memory synapses, thereby stabilizing these synapses (Figure 1). This would predict that new synapses have an increased ability to form memory synapses compared to other synapses, but that memories are distributed between old and new synapses. Importantly, it also predicts that memories made after a single trial would initially be mainly stored at old synapses, even if the stimulus induced the formation of new synapses. Recruitment of the new synapses would require additional learning trials, or perhaps reinforcement by recall during sleep.

### Are memory synapses identifiable by changes in spine morphology?

A correlate of LTP is structural-LTP where spine size increases after stimulation and is maintained in a protein-synthesis dependent manner (Tanaka et al., 2008; Bosch and Hayashi, 2012). While initial increases in the size of the synapse are not necessarily linked to increases in the size of the post-synaptic density (PSD), at later stages increases in the size of the PSD are also seen, linking structural LTP to increases in synaptic strength (Bosch et al., 2014). While there are numerous studies showing structural LTP in slices and dissociated neurons, there are few documented increases in spine size *in-vivo* after learning. One particularly striking example used a reporter consisting of several PSD domains from PSD-95 fused to a photo-activatable Rac and synthesized locally through inclusion of the 3′untranslated region of Arc in the construct (Hayashi-Takagi et al., 2015). The protein produced from the construct localized specifically to synapses that supported memory, as shown by the loss of memory after photo-activation of Rac and shrinkage of these synapses (Hayashi-Takagi et al., 2015). Activation of Rac had previously shown to be implicated in active forgetting (Shuai et al., 2010). Synapses containing this construct consisted of both new synapses and synapses whose size increased after learning providing evidence for increases in spine size occurring at putative memory synapses (Hayashi-Takagi et al., 2015). The concomitant shrinking of spine size and erasure of memory by photo-activating Rac was seen 1 day after memory formation, but not at later times (Hayashi-Takagi et al., 2015). It was not clear from this study whether this was due to synapses becoming insensitive to Rac activation after formation of a long-lasting molecular memory complex, or to the loss of the construct from the putative memory synapses. It was also not clear whether only activated synapses were affected by Rac (i.e., a specific memory complex is sensitive to Rac) or whether specificity resulted from the localization of the construct at only activated synapses. Interestingly, the construct's localization at active synapses was mediated mainly through stabilization of the construct at the activated synapses since specific localization was lost in the presence of a proteasome inhibitor (Hayashi-Takagi et al., 2015). Stabilization of persistent protein kinases has also been proposed to play a role in the localization of these proteins to activated synapses (Hu et al., 2017b).

## What are the molecular complexes that define memory synapses?

There are many molecular complexes that are known to differentiate synapses from one another. We will focus on excitatory synapses and the AMPA receptors that are a major target for many forms of synaptic plasticity thought to underlie memory. It is highly likely that memories are also made through strengthening specific inhibitory synapses (Lucas and Clem, 2017) through trafficking and diversity of GABA receptor complexes (Mele et al., 2016), but this is outside the scope of this review. Moreover, while this review focuses on AMPA receptors, there are numerous examples where plasticity also affects the number and/or composition of NMDA receptors at synapses (Grosshans et al., 2002; Prybylowski et al., 2005; Harney et al., 2008; Kwon and Castillo, 2008). However, since the majority of the EPSP (the measure of synaptic strength) is encoded by AMPA receptors, the changes in NMDA receptors are presumably an important mediator of metaplasticity, not the direct encoding of the memory trace and thus the following discussion focuses on AMPA receptors. It is certainly possible, however, that at some specialized synapses, insertion of specific NMDA receptor complexes can also participate in forming a molecular complex encoding the memory trace.

### Diversity of AMPA receptor complexes

There are at least five sources of diversity in AMPA receptor complexes: (1) Separate genes encode distinct isoforms of AMPA receptors (there are four AMPA receptors in vertebrates, GluA1-4); (2) Post-transcriptional modifications of these receptors, including alternative splicing (Penn and Greger, 2009) and RNA editing (Hood and Emeson, 2012) which can generate many different forms of the proteins even from the same transcribed mRNA (Greger et al., 2017); (3) The tetrameric structure of the receptors allows multiple combinations of distinct genes and post-transcriptionally modified genes to combine, increasing the complexity of a single tetrameric receptor (Henley and Wilkinson, 2016); (4) Post-translational modifications such as phosphorylation (Lu and Roche, 2012; Wang et al., 2014), ubiquitination (Goo et al., 2015), acetylation (Wang et al., 2017), and palmitoylation (Han et al., 2015) alter the function of the receptors; and (5) Distinct associated proteins make up distinct AMPA receptor complexes, such as TARPS, Cornichons, and others (Schwenk et al., 2012). Since a large amount of synaptic plasticity rests on transferring AMPA receptors in and out of the postsynaptic density, it does not take much imagination to see that depending on the composition of these AMPA receptor complexes, the regulation of their insertion and removal at synapses will be different. I propose that distinct AMPA receptor complexes are specialized for inclusion at memory synapses.

### Memory synapses are defined by rules of AMPA receptor trafficking that in turn are defined by distinct AMPA receptor complexes

The role of persistent protein kinases in stabilizing increases in synaptic strength in the postsynaptic neuron depends on their ability to stabilize AMPA receptor complexes by preventing their endocytosis. The evidence supporting this postulate is that the loss of memory seen after transiently inhibiting the kinases with ZIP can be blocked if endocytosis of AMPA receptors is concomitantly blocked (Migues et al., 2010; Dong et al., 2015). Moreover, increasing AMPA receptor endocytosis may play a role in active forgetting, the flip side of memory erasure (Migues et al., 2016). A large missing piece of this model is the identification of the phosphorylation sites used by the persistent protein kinases that are important to block endocytosis.

In general, the stability of an AMPA receptor complex at the synapse depends on two aspects of the complex. First, the AMPA receptor complex requires interaction with a scaffold protein that anchors it to the postsynaptic density. These scaffold proteins, such as PSD-95 that connects to AMPA receptors through the TARP proteins, and GRIP, which connects to the PDZ binding site on GluA2, are known modulators of AMPA receptor stability at the synapse (Ehrlich and Malinow, 2004; Lu and Ziff, 2005). Second, the AMPA receptor complex connects to the endocytic machinery. Stimuli that enhance binding of AMPA receptors to proteins like PICK, HIP1 and Arc that connect the AMPA receptor to the endocytic machinery lead to destabilization of AMPA receptors at the synapse (Xia et al., 2000; Metzler et al., 2003; Lu and Ziff, 2005; Chowdhury et al., 2006; Fiuza et al., 2017; Wall and Correa, 2017). Some AMPA receptor subunits interact directly with the endocytic machinery, such as the AP2 binding site in GluA2 (Kastning et al., 2007). Interestingly, the ARF guanine exchange factor, BRAG2, binds to a tyrosine motif in GluA2/3 (Scholz et al., 2010). The peptide encoding this tyrosine motif was the peptide used to block memory loss after application of ZIP (Migues et al., 2010; Dong et al., 2015), suggesting that inhibition of BRAG2 may be a target of persistent kinase phosphorylation (Sacktor, 2011). However, since many forms of LTD require BRAG2 (Scholz et al., 2010), the specific removal of AMPA receptor complexes at memory synapses may also require distinct motifs or adaptors that are regulated by persistent kinase activity in addition to BRAG2. Overall, since distinct AMPA receptor complexes interact differentially with stabilizing scaffold proteins and endocytic machinery adaptors, these distinct complexes should be capable of being differentially removed by distinct stimuli. One would predict that an AMPA receptor complex important for memory should be removed by specific manipulations that erase memory (such as ZIP or reactivation of memory) while other AMPA receptor complexes at non-memory synapses would be resistant to these manipulations.

It is known that AMPA receptor complexes are differentially sensitive to different stimuli. For example, NMDA-dependent LTD and mGLUR-LTD appear to act on separate AMPA receptor complexes since these LTDs do not occlude each other (Huber et al., 2001; Connelly et al., 2011). Whether either of these forms of LTD remove “memory” AMPA receptor complexes is not clear. It has been suggested that mGLUR-LTD only works on synapses that have previously undergone LTP (Jones, 2017), implying that this form of LTD may act to remove memory synapses. In contrast, optogenetic stimulations more similar to stimuli that activate NMDA receptor-dependent LTD have been shown to erase memories (Nabavi et al., 2014; Kim and Cho, 2017), suggesting that AMPA receptor complexes at memory synapses are sensitive to this type of stimulation.

Up to this point, we have discussed the synapse connecting two neurons as a unified whole. However, super-resolution microscopy has revealed that synapses are made up of modules, where a cluster of AMPA receptors coupled to scaffolds is aligned with a presynaptic release site defined by proteins such as RIM or UNC13 (MacGillavry et al., 2013; Nair et al., 2013; Tang et al., 2016; Lisman, 2017b). Each synapse contains a number of these modules. It is possible that, instead of a “memory synapse” there are “memory modules” at a synapse that are sensitive to disruption by blockade of persistent protein kinases Thus, the connection between two neurons may consist of a stable component and a memory component. Removal of either component may be sufficient to reduce synaptic strength and thus compromise memories that depend on this increase in synaptic strength. The question then becomes, is it the stable or the memory component that is removed by various forms of LTD.

### Role of synaptic adhesion molecules in defining a slot

So far we have focused on the postsynaptic molecular memory complex, but the work in *Aplysia* suggests important complexes in the presynaptic neuron that can also be interfered with to erase memory. Memory synapses or memory modules at synapses may be defined through specific trans-synaptic adhesive interactions that align the AMPA receptor complexes specific for memory with specific presynaptic molecular complexes. These presynaptic molecular complexes may also have specializations important for memory, including the ability to be disrupted by inhibitors of persistent kinases. Indeed, if the target of the persistent kinases is the stability of the trans-synaptic adhesion proteins, then the ability to erase the memory through dominant negative PKMs in either the presynaptic or postsynaptic cell would be explained (Hu et al., 2017a). A number of presynaptic-postsynaptic pairings important for synapse formation and stabilization have been identified (Fogel et al., 2007; Gottmann, 2008; Choi et al., 2011; Kriebel et al., 2011; Südhof, 2012; Enneking et al., 2013). It has been established that these presynaptic-postsynaptic pairings can define the properties of the synapse. Probably the best example of synaptic connections being determined by presynaptic-postsynaptic pairing is a specialized synapse on the axon hillock directed by neurofascin/neuropilin interactions (Telley et al., 2016). Broad knockouts of neurexin/neuroligins also have distinct effects on different synapses, suggesting a model where multiple specializations of synapses are due to specific pairings of neurexin/neurligin splice forms (Zhang et al., 2015; Chen et al., 2017). Indeed, *Aplysia* long-term facilitation, but not basal synaptic strength, is blocked by decreasing levels of neurexin or neuroligin, suggesting a specific role for this adhesive pair at memory synapses in this system (Choi et al., 2011). It should be emphasized again that there is not one universal molecular complex that defines memory. As discussed earlier, even at the simple sensory-motor neuron synapse of *Aplysia*, at least two distinct memory complexes that are erased by distinct manipulations can form (Hu et al., 2017b). Thus, there may be multiple adhesion pairs that define distinct types of memory synapses or memory modules at synapses.

One can envision trans-synaptic adhesion as defining what is often referred to in the plasticity literature as a “slot.” A “slot” on the postsynaptic side would attract specialized AMPA receptor complexes for memory. If slots exist, then memory erasure must not only remove AMPA receptors, but the slot as well, else memory would recover after the inhibition of persistent protein kinases fade. One of the most provocative findings about memory erasure by persistent kinase inhibitors is that blockade of endocytosis can stop the erasure from occurring. The simple explanation for this result is that it is the endocytosis of AMPA receptors prevented by the persistent protein kinases, since the inhibitors of endocytosis used in these experiments is from the structure of the C-terminal of the GluA2 receptor. However, the actions of these inhibitors are presumably at an endocytosis adaptor, such as BRAG2, that may have other substrates as well. An appealing model would be that as well as causing endocytosis of the AMPA receptors, the “slot” proteins would also be a target of endocytosis after inhibition of the persistent protein kinase. This would be true in the presynaptic cell as well as inhibition of persistent kinases in the presynaptic cell could also lead to endocytosis of the presynaptic partner of the adhesion complex.

## What would constitute proof of a “memory synapse”

In this review, I have presented arguments supporting the idea that synapses that encode memory, as long as they are sensitive to specific erasure, have distinct molecular components. If this is true, the identification of these molecular components should allow the identification of memory synapses by live imaging techniques and immunocytochemistry. Moreover, these synapses or synaptic modules should be uniquely sensitive to disruption by memory erasure, either by reconsolidation or through the pharmacological and genetic techniques that have been shown to erase behavioral memory. Until such evidence is provided, the concept of a memory synapse remains an unproven hypothesis.

## Putting it all together

**Figure 2:**
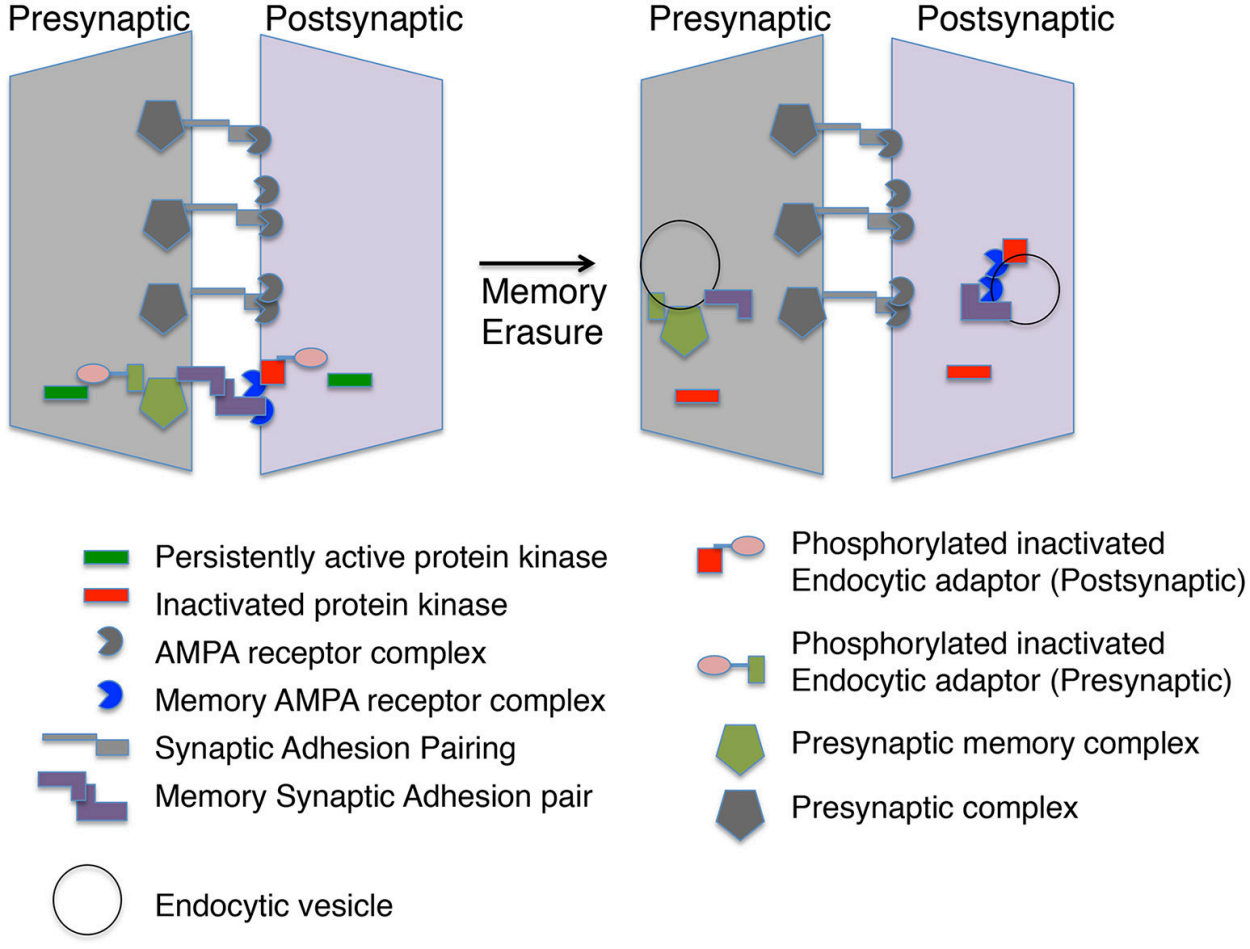
A memory module at a synapse. The molecular memory complex is portrayed as a module with basal synaptic complexes at one synaptic connection. The molecular memory complex has memory-specific synaptic adhesion proteins, AMPA receptor complexes and presynaptic complexes. A persistent protein kinase is continually phosphorylating an endocytic adaptor protein preventing endocytosis of the molecular memory complex. Inactivation of the persistent protein kinase leads to endocytosis of the molecular memory complex in both the presynaptic and postsynaptic neuron.

Generating a memory synapse may require multiple rounds of modifications (Figure 1). Persistent protein kinases need to be formed and stabilized. Both pre-and postsynaptic gene expression may be required to generate specific adhesion proteins that attract specific AMPA receptor complexes and presynaptic specializations, which are stabilized by the persistent protein kinases (Figure 2). Mechanisms for replacement of all these molecular parts over time are also required and this may require persistent transcriptional marks in Engram neurons. Yet, the entire idea of a memory synapse rests on its lability, and the ability of the animal to modify its model of the world through new information. Thus, there must be an ability to erase these structures when their representation of the world is no longer correct. We have made progress defining some of the putative players, such as PKMs and AMPA receptor complexes, but there are many missing players awaiting identification, including specific scaffolds, endocytic adaptors, substrates for persistent protein kinases, and specific transsynaptic adhesion pairs. By reviewing the possible molecular markers of memory synapses and speculating on what markers may exist, we hope to stimulate research in this field. Identifying the missing pieces will help to anchor the concept of memory synapses on a stronger scaffold of molecular findings.

## Author contributions

The author confirms being the sole contributor of this work and approved it for publication.

### Conflict of interest statement

The author declares that the research was conducted in the absence of any commercial or financial relationships that could be construed as a potential conflict of interest.
